# Supplementary material for: Dark Diazotrophy during the Late Summer in Surface Waters of Chile Bay, West Antarctic Peninsula
Source: Microorganisms. 2022 May 31;10(6):1140. doi: 10.3390/microorganisms10061140 (PMC9227844; doi:10.3390/microorganisms10061140)
Supplement: Supplementary file 1 [file microorganisms-10-01140-s001.zip › microorganisms-1526919-supplementary.pdf]

Table S1. Chemoautotroph organisms in *nifH* phylogenetic tree related with diazotrophs from Chile Bay

Organism	Family	Metabolic class	Cluster	Reference	Percentage of identical matches*	e-value*
Methylocapsa palsarum	*Beijerinckiaceae*	Methanotroph	1	[112]	93.39%	3.40E-84
Methyloceanibacter sp. wino2	*Hyphomicrobiaceae*	Methylotroph	1	[113]	93.39%	1.53E-84
Methyloceanibacter stevinii	*Hyphomicrobiaceae*	Methanotroph	1	[114]	92.56%	5.74E-84
Methylocystis bryophila	*Methylocystaceae*	Methanotroph	1	[115]	95.04%	1.51E-84
Methylocystis sp. SC2	*Methylocystaceae*	Methanotroph	1	[116]	97.52%	2.10E-87
Xanthobacter autotrophicus Py2	*Xanthobacteraceae*	Methylotroph	1	[117]	95.87%	1.26E-86

*Values of BLASTP against the Chile Bay NifH reads.
